# Hierarchical Task-Parameterized Learning from Demonstration for Collaborative Object Movement

**DOI:** 10.1155/2019/9765383

**Published:** 2019-12-02

**Authors:** Siyao Hu, Katherine J. Kuchenbecker

**Affiliations:** ^1^Department of Mechanical Engineering and Applied Mechanics and GRASP Laboratory, University of Pennsylvania, Philadelphia 19104, USA; ^2^Haptic Intelligence Department, Max Planck Institute for Intelligent Systems, 70569 Stuttgart, Germany

## Abstract

Learning from demonstration (LfD) enables a robot to emulate natural human movement instead of merely executing preprogrammed behaviors. This article presents a hierarchical LfD structure of task-parameterized models for object movement tasks, which are ubiquitous in everyday life and could benefit from robotic support. Our approach uses the task-parameterized Gaussian mixture model (TP-GMM) algorithm to encode sets of demonstrations in separate models that each correspond to a different task situation. The robot then maximizes its expected performance in a new situation by either selecting a good existing model or requesting new demonstrations. Compared to a standard implementation that encodes all demonstrations together for all test situations, the proposed approach offers four advantages. First, a simply defined distance function can be used to estimate test performance by calculating the similarity between a test situation and the existing models. Second, the proposed approach can improve generalization, e.g., better satisfying the demonstrated task constraints and speeding up task execution. Third, because the hierarchical structure encodes each demonstrated situation individually, a wider range of task situations can be modeled in the same framework without deteriorating performance. Last, adding or removing demonstrations incurs low computational load, and thus, the robot's skill library can be built incrementally. We first instantiate the proposed approach in a simulated task to validate these advantages. We then show that the advantages transfer to real hardware for a task where naive participants collaborated with a Willow Garage PR2 robot to move a handheld object. For most tested scenarios, our hierarchical method achieved significantly better task performance and subjective ratings than both a passive model with only gravity compensation and a single TP-GMM encoding all demonstrations.

## 1. Introduction

Many modern humanoid robots are designed to operate in human environments, like homes and hospitals. If designed well, such robots could help humans accomplish tasks and lower their physical and/or mental workload. One particularly interesting task type is jointly manipulating an object with a partner [1], as it requires human collaboration, shared physical control, and adapting to new situations. The way in which one creates new robot behaviors or updates known behaviors should be intuitive and natural so that users who are not familiar with robotics can easily customize the robot to their specific environment and needs. This research is aimed at designing an intelligent robot controller that achieves these desired characteristics.

As opposed to having an operator devise control policies and reprogram the robot for every new situation it encounters, learning from demonstration (LfD, also known as programming by demonstration (PbD)) provides a direct method for robots to learn and replicate human behaviors [2, 3]. LfD control policies are learned from demonstrations in which a human teacher controls the robot to accomplish the task. Various learning algorithms are suitable for encoding interactions recorded during demonstrations, such as hidden Markov models (HMMs) [4] and hidden semi-Markov models (HSMMs) [5]. By extending the HSMM framework, Rozo et al. enabled the robot to be proactive if the partner does not follow the demonstrations, which were encoded by the observed temporal patterns and sequential information [6]. Dynamic motion primitives (DMPs) [7] provide another framework for interaction encoding, for example, learning an adaptive, sensor-driven interaction between two coupled agents [8]. Instead of learning or placing basis functions for the forcing term, Pervez et al. presented a DMP-based method that accommodates spatial and temporal variations in demonstrations, different initial and final conditions, and partial executions by directly encoding the phase variable and forcing term value in a Gaussian mixture model (GMM) and synthesizing the forcing term at test time using Gaussian mixture regression (GMR) [9].

Another promising learning framework is using a GMM and GMR directly on the demonstrated trajectories, where multiple channels of information (e.g., position and velocity of the robot gripper) are encoded jointly by a GMM. The conditional probability density function of the outputs on the inputs can be calculated and used in GMR for a wide range of applications such as trajectory retrieval [10]. The task-parameterized GMM (TP-GMM) framework utilizes task parameters to annotate demonstrations, and it allows generalization to undemonstrated situations by manipulating the demonstrated data with respect to the undemonstrated task parameters [11]. Rozo et al. used the TP-GMM framework for human-robot collaborative tasks, additionally modeling the robot dynamics with an impedance model that has unit mass and constant damping [12]. In a similar spirit, Pervez and Lee developed task-parameterized DMP (TP-DMP) to include such task parameters in a mixture of GMMs [13], extending the GMM that previously encoded only the phase variable and the forcing term value in [9].

In addition to utilizing various learning frameworks, LfD approaches create the opportunity for the robot to determine when new demonstrations are needed, thus avoiding poor or even dangerous actions. Uncertainty in generalized trajectories or cost functions has been used as a trigger for requesting demonstrations, where uncertainty can be calculated from, e.g., Query by Bagging [14] or a Gaussian Process (GP) [15]. Chernova and Veloso used confidence in execution to detect unfamiliar or ambiguous states that require new demonstrations [16]. In another approach, a GMM gating model that is based on observed human motions determines whether the test task is likely to be contained in the Interaction ProMPs that the robot has already learned or whether new demonstrations are necessary [17]. In these approaches, the new test is compared to what the robot has experienced, and new demonstrations are requested when the robot deems it necessary. On the other hand, Abi-Farraj et al. considered generalized trajectories for refining the learned distribution via an information gain threshold so that the robot does not need to request additional demonstrations [18].

Many approaches are aimed at building skill libraries from demonstrations. For example, Muelling et al. learn a library of DMPs from demonstrations for table tennis, and at test time, the output control policy is a weighted average of the DMP skills generated from a kernel function on input stimuli (hitting position and velocity) and weight parameters that prioritize certain skills obtained using reinforcement learning [19]. Since a weighted average was used as a means for generalization, predicting task performance for tests was difficult, i.e., the combination of a set of good demonstrations may not necessarily result in good behavior, and thus, reinforcement learning was necessary to prune or prioritize certain skills in the DMP library [19]. As a result, when a new motion primitive is added or an existing one is deleted, the library may need to be retrained for it to converge again. In contrast, online learning is achieved in [17] by incrementally building the GMM gating model and the Interaction ProMPs, while the gating model selects only one most likely model for generalization. Other examples that incrementally build skill libraries from new demonstrations include [20–22]. Existing methods that incrementally train GMMs have also been adapted for TP-GMMs [23].

In this paper, we propose a hierarchical framework that considers the three aspects of LfD mentioned above. First, we utilize the TP-GMM algorithm [11] as the basis for demonstration encoding and generalization because of its validated merits in many tasks. Second, our utility functions operate on task parameters and allow the robot to determine when to request new demonstrations. Third, we incrementally build a library of TP-GMMs to continuously improve test performance as new demonstrations become available.

Our approach is most similar in spirit to [13, 17]. Akin to [13], we build a single skill (GMM) from one or more demonstrations corresponding to the same task parameters. We also aim to learn from a small number of available demonstrations and overcome the sparsity of task parameters in training data, although our approach uses a gating function (as does [17]) to choose the most applicable skill for generalization with TP-GMM instead of mixing skills together [13]. Consequently, it is possible to determine when new demonstrations are required in both our approach and [17] but not in [13], and our approach can incrementally build the skill library as new demonstrations become available, while [13] learns from batch data. Another important difference between our approach and [13] is that because task parameters are encoded in GMMs and their numerical differences determine the relative contributions of the mixture [13], it is not straightforward to include orientation of relevant objects/frames in the task parameters: for example, a Euler angle of 0.9*π* rad is numerically closer to 0.5*π* rad than to −0.9*π* rad, but one could expect the skill from −0.9*π* rad to be more relevant when extrapolating to 0.9*π* rad.

As for [17], demonstrations are represented by linear combinations of basis functions, and the weights are encoded in the GMM. As a result, each demonstration contributes only one data point to the GMM training procedure, and the dimensionality of the weight space depends on the number of basis functions, which may need to be high to have enough expressiveness in constructing the trajectories; these two factors combined may make it necessary to acquire a large number of demonstrations to avoid overfitting. In addition, task parameters are not considered in [17], and thus, this approach is more suitable when distinguishing and performing multiple types of tasks, e.g., if the robot needs to hand over objects and help humans stand up. In contrast, our approach and [13] consider different instances of the same task type and are aimed at improving generalization performance when, e.g., the position of the human receiving the object changes. Finally, our approach utilizes TP-GMM directly on trajectories, so it is possible to include the position, velocity, time, and/or force dimensions in the model depending on the task, making our approach more versatile than the trajectory retrieval functions in [13, 17].

**Figure 1:**
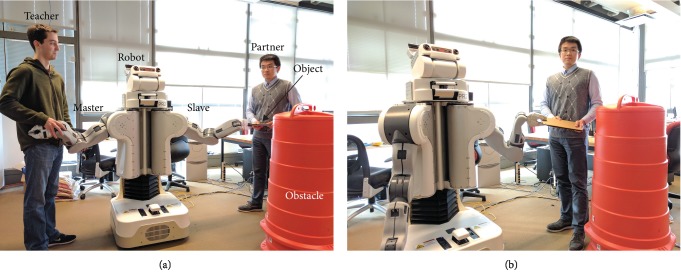
Training and testing scenarios for collaborative manipulation with a Willow Garage PR2. (a) The teacher teleoperates the robot to manipulate an object with the partner. (b) The robot collaborates with the partner during evaluation.

The main contributions of this work are a hierarchical LfD structure of task-parameterized models for object movement tasks, as well as analysis of the generalization performance of TP-GMM and the proposed hierarchical framework both in simulation and on real hardware. The simulated movement task shows that our hierarchical structure can predict test performance via a utility function that measures task situation similarity, improve generalization performance, and reduce computational load during training. The real movement task shows that a robot controlled by the proposed hierarchical structure collaborates with human subjects more effectively than TP-GMM or a passive robot. We focus on an object movement task, such as the scenario shown in Figure 1, because it is ubiquitous in everyday life and could benefit from robot assistance.

## 2. Materials and Methods

In this section, we first briefly introduce the TP-GMM algorithm [11] and use a simplistic task to illustrate potential issues with its typical implementation. Then, we introduce the proposed hierarchical structure that utilizes TP-GMM, which we instantiate and compare against typical TP-GMM in a simulated movement task. Finally, we detail the human-subject study where naive users collaboratively manipulated an object with a Willow Garage PR2 and evaluated its performance with the typical TP-GMM algorithm, the proposed hierarchical structure, and passive gravity compensation.

### 2.1. Overview of the Task-Parameterized Gaussian Mixture Model Algorithm

We use TP-GMM [11] to encode demonstrations and generate controller commands during test time. TP-GMM has been used to enable a robot to learn collaborative beam lifting [24] as well as object transportation and chair assembly [12]; its typical implementation, which we call vanilla TP-GMM (VT), has shown good generalization capabilities in these applications.

In the following sections, we use task situations to denote particular instances of a task: for example, in the object-moving task, moving from point A to point B and moving from point A to point C are two different task situations. In the context of TP-GMM, the task parameters fully define a task situation.

#### 2.1.1. The TP-GMM Algorithm

The *n*th demonstration (*n* = 1, 2, ⋯, *N*) contains *L*_*n*_ data points ({**ξ**_*n*,*l*_}_*l*=1_^*L*_*n*_^), and each data point may have dimensions of time, position, velocity, etc., at a given time step. The task parameters (**p**) are defined as *P* affine transformations ({**A**_*p*_, **b**_*p*_}_*p*=1_^*P*^) that include information about the task situation (e.g., poses of the start and goal frames). In addition, task parameters need to be compatible with the data; for example, if each data point contains the instantaneous 3D Cartesian position and velocity of the robot gripper, i.e., **ξ** = [**x**^⊤^ **v**^⊤^]^⊤^, one can define

(1) $$\begin{array}{c} A_{p}=\left[\begin{array}{cc} R_{p} & 0\\ 0 & R_{p} \end{array}\right]\; \end{array}$$

and $b_{p}={\left[\begin{array}{cc} r_{p}^{⊤} & 0 \end{array}\right]}^{⊤}$, where **R**_*p*_ and **r**_*p*_ represent the orientation and position of the *p*th relevant reference frame so that matrix operations such as **A**_*p*_^−1^(**ξ** − **b**_*p*_) are valid and physically meaningful.

Algorithm 1 includes a brief overview of the TP-GMM algorithm. The training step contains only the ENCODE function, which transforms data points into each task frame and then fits a TP-GMM **Π** = {*π*_*m*_, {**μ**_*m*_^(*p*)^, **Σ**_*m*_^*p*^}_*p*=1_^*P*^ }_*m*=1_^*M*^, where *M* is the number of Gaussian clusters, *π*_*m*_ is the mixture coefficient, and {**μ**_*m*_^(*p*)^, **Σ**_*m*_^*p*^} are the Gaussian mean and covariance matrices of the *m*th cluster in the *p*th task frame. The FITGMM function is close to the standard procedure in fitting a GMM with expectation maximization. The testing step includes two functions: DECODE transforms each Gaussian cluster in **Π** according to the test task parameters and generates a regular GMM **π** in the global frame, and GMR computes a trajectory for the test situation, which can be used as controller commands. For example, the output **ξ**^*ℴ*^ could be the velocity and the input **ξ**^*𝒾*^ the position of a robot gripper, i.e., during automatic execution, the robot could derive the desired velocity given its current position.

TP-GMM exploits locally consistent features among demonstrations in each task frame (ENCODE) and transforms them according to new situations (DECODE), generally yielding reliable performance for both interpolation and extrapolation in many applications [12, 24].

However, TP-GMM does not have an explicit estimate of how well the local information would perform with respect to the new task situation in GMR; therefore, it cannot determine when new demonstrations are necessary. For example, it may have poor generalization when the new task situation is too different from what has been demonstrated. In addition, GMR may have trouble even when the test situation is exactly the same as a demonstrated one, depending on the quality and consistency of the demonstrated data. These potential issues are illustrated in the next section.

#### 2.1.2. TP-GMM with a Simplistic Task

**Figure 2:**
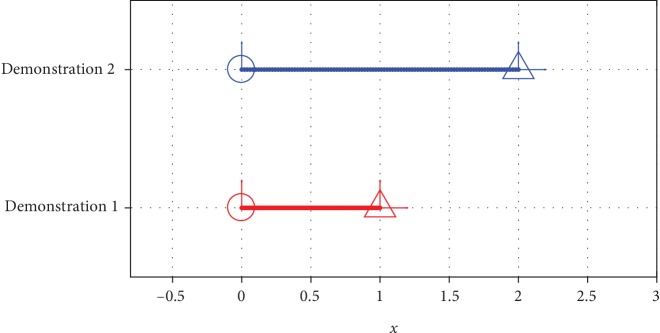
Demonstrated trajectories for a simplistic movement task. Circles represent the start positions, and triangles represent the goal positions of the task situations.

Suppose the task of interest is moving from a known start position to a known goal position on a one-dimensional line, and the strategy that generates demonstrations uniformly connects the start and goal points in 100 time steps, as shown in Figure 2. In this example, data points contain $\xi ={\left[\begin{array}{cc} t & x \end{array}\right]}^{⊤}$, where *t* = 1, 2, ⋯, 100 is the time step and *x* is the coordinate. Consequently, we include the start and the goal task frames for task parameters when training a TP-GMM, and for each, we have **A** = **I**_2_ and $b={\left[\begin{array}{cc} 0 & r \end{array}\right]}^{⊤}$, where *r* represents the location of the frame. Without loss of generality, the start positions of all task situations are at *x* = 0.

**Figure 3:**
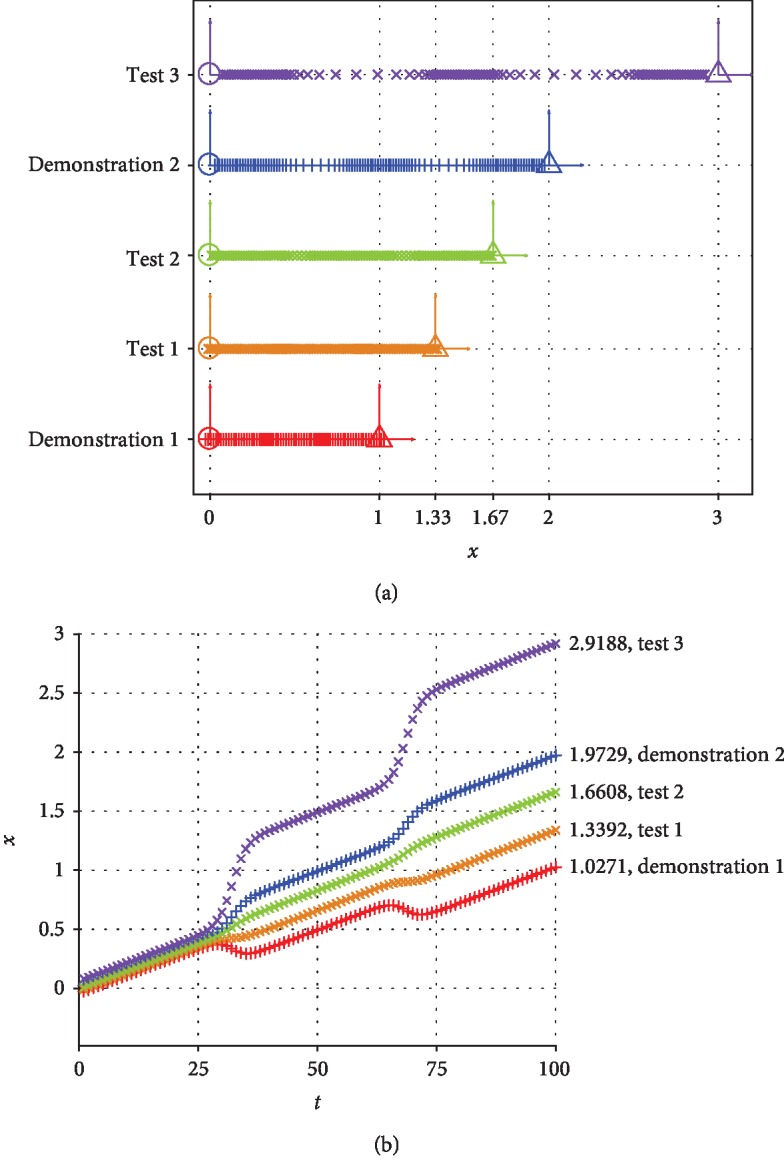
Generalized trajectories for the two demonstrated situations and three new test situations using vanilla TP-GMM. (a) Generalized trajectories. (b) Generalized trajectories over time.

Using vanilla TP-GMM with *M* = 3 in Algorithm 1, we can generalize trajectories for different test situations (**ξ**^*𝒾*^ = *t* and **ξ**^*ℴ*^ = *x*), as shown in Figure 3. We use three clusters in this simulation because they generally cover the trajectories well and require little time to train, but other numbers work as well.

It can be seen that the generalized trajectories cover the distance between the start and goal positions well (Figure 3(a)), but they no longer contain uniform step lengths (Figure 3(b)). If the start and goal are close to each other (e.g., demonstration 1 in Figure 3), the generalized trajectory may even reverse the direction of motion at time values around 30 and 70, where the dominant Gaussian cluster changes. On the other hand, if the start and goal are far from each other (e.g., test 3 in Figure 3), the generalized trajectory may cause very large velocities near the same time points. Inconsistencies such as reversed motion direction and high magnitude velocity may cause instabilities on real hardware and might seem like faulty behaviors to a naive user. Another inconsistency is that the generalized first and last trajectory points do not always align with the prescribed start and goal positions, as shown in Figure 3(b), which may cause jumps at the beginning and end of autonomous execution. Importantly, although the types and locations of the inconsistencies can vary in different implementations, their existence is not specific to the task or the number of clusters used in simulation.

What causes this difficulty with generalization? TP-GMM can be viewed as being similar to a regression algorithm: task parameters are the independent variables, and trajectories are generalized from local information in each task frame. Consequently, the exact information from each individual training point may be lost (e.g., demonstration 1 in Figure 3). In addition, because the function that maps task parameters to trajectories is highly nonlinear, the regression model does not have enough information to accurately generalize for a test point outside of the trained region (e.g., test 3 in Figure 3). Vanilla TP-GMM can have good generalization properties (test 2 in Figure 3), but it does not offer an estimate of generalized performance and thus cannot differentiate between tests 1, 2, and 3. Instead, it has to rely on the robot controller to handle the potential peculiarities of the generated trajectory. Additionally, because information from demonstrations is stored locally with respect to each task frame, the original global strategy (in this case, connecting start and goal positions with uniform step lengths) is largely lost after modeling in TP-GMM.

### 2.2. Hierarchical Task-Parameterized Learning from Demonstration

To preserve the powerful generalization capability of TP-GMM and overcome the previously discussed shortcomings, we propose a hierarchical structure (HS) that explicitly reasons about task parameters using three utility functions in the following steps.

First, we define a distance function that operates on a pair of task situations and outputs a scalar value, representing how similar the two situations are to each other. We argue that this scalar value can serve to estimate test performance, which can then be a trigger for requesting demonstrations. Second, sets of demonstrations associated with the same task parameters are each encoded as their own TP-GMM. Third, given a test situation, we use the distance function to select the TP-GMM from only the most similar situation. In addition, with only one situation per TP-GMM, we can manipulate the Gaussian clusters to enhance generalization with two morphing functions, because the exact information and strategy from that individual training data set are preserved. Finally, the hierarchical structure makes it straightforward to encode a large variety of task situations in the same framework.

This section empirically validates the distance function as a test performance estimator, the steps in the proposed hierarchical structure, and the improvements of our approach compared to vanilla TP-GMM for a simulated movement task.

#### 2.2.1. Simulated Task Definition

**Figure 4:**
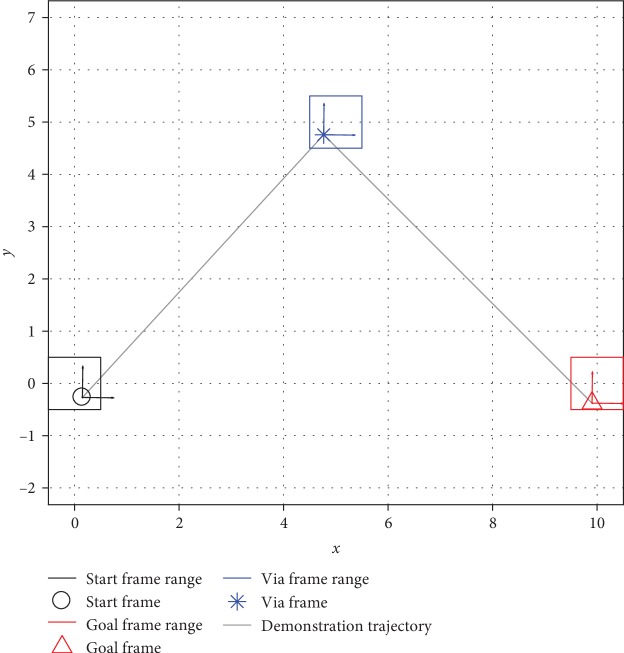
An example task situation and its trajectory. The frame ranges show the regions from which each task frame is randomly sampled.

We use a movement task with three task frames, representing the start (**R**_1_,  **r**_1_), goal (**R**_2_,  **r**_2_), and via (**R**_3_,  **r**_3_) points on a two-dimensional plane. Task parameters for these frames include rotation matrices with *x*-axes parallel to a vector pointing from the start to the goal, *z*-axes pointing out of the page, and *y*-axes following the right-hand rule. The demonstration strategy uniformly connects the start and via points using a straight line with 100 time steps and then uniformly connects the via and goal points using a straight line with another 100 time steps ($\xi ={\left[\begin{array}{ccc} t & x & y \end{array}\right]}^{⊤}$, where *t* = 1, 2, ⋯, 200), as shown in Figure 4. We call a unique specification of the frame ranges a *task configuration*.

We use this example task to instantiate the utility functions and the evaluation procedure in the following subsections. Nevertheless, the hierarchical structure can also be used in other tasks and/or with different data dimensions (such as in Section 2.3).

#### 2.2.2. Distance Function

For the example task, we define the distance function in Algorithm 2 for two task situations (**p**_*A*_ and **p**_*B*_): the task frames of the compared situations are transformed into their start frames, and the distance function value is calculated as the sum of squares of the distances between the corresponding goal and via points. Note that there may be many possible definitions for the distance function; for example, one could also choose to include a norm on the rotation matrices. Our particular definition builds on the understanding that TP-GMM aligns clusters in each task frame, and thus, the task frames' positions with respect to each other are more important than their absolute positions in the world frame.

To understand the distance function, consider the two-frame task in Section 2.1.2, where Algorithm 2 would simply calculate the square of the distances between the goals of each situation. In particular, DISTANCE(**p**_test,3_, **p**_demo,1_) = (3 − 1)^2^ = 4, while DISTANCE(**p**_test,3_, **p**_demo,2_) = (3 − 2)^2^ = 1. Therefore, test 3 would be considered more similar to demonstration 2 than it is to demonstration 1.

#### 2.2.3. Situation and GMM Morphing

**Figure 5:**
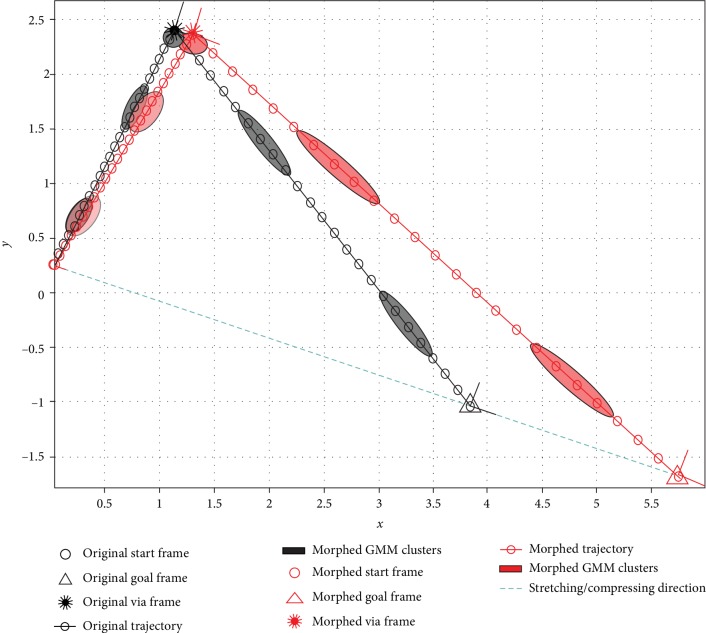
Illustration of Equations (2), (3) and (4) and the morphing functions in Algorithm 3. The original task frames are randomly sampled, and the original trajectory and GMM clusters are generated from the demonstration strategy and trained accordingly. The morphed frames, trajectory, and GMM clusters are calculated from the original ones with *α* = 1.5. Note that the original and morphed start frames coincide.

Similar to using DMP on generalized trajectories from a GP to ensure that the prescribed goals are reached [15], we introduce a generalization-enhancing strategy that is specific to the movement task: if the start and goal points become farther or closer to each other, then the trajectory can be proportionately stretched or compressed in the start-goal direction to accommodate the change:

(2) $$\begin{array}{c} T=\frac{\left(r_{2}-r_{1}\right){\left(r_{2}-r_{1}\right)}^{⊤}}{{\left(r_{2}-r_{1}\right)}^{⊤}\left(r_{2}-r_{1}\right)}, \end{array}$$

(3) $$\begin{array}{c} r={\left[\begin{array}{cc} x & y \end{array}\right]}^{⊤}, \end{array}$$

(4) $$\begin{array}{c} r^{\prime }=r_{1}+\alpha T\left(r-r_{1}\right)+\left(I-T\right)\left(r-r_{1}\right)=\left(I+\alpha T-T\right)r-\left(\alpha -1\right)Tr_{1}, \end{array}$$

where **T** is the projection operator along a unit vector from start (**r**_1_) to goal (**r**_2_), **r** is an arbitrary point of the original trajectory, *α* is the scalar value representing the extent of stretching or compression, and **r**′ is the proportionately changed new trajectory point, as shown in Figure 5.

Consequently, we can generate task parameters and Gaussian clusters for the new situation in the same manner.  Algorithm 3 details how this process can be carried out in accordance with the current definition of $\xi ={\left[\begin{array}{ccc} t & x & y \end{array}\right]}^{⊤}$ (see definitions of **T**′ and **T**^″^), where **T** is defined in Equation (3). The morphed task situation and GMM clusters are plotted in Figure 5.

#### 2.2.4. Hierarchical Structure

With the utility functions defined in Algorithms 2 and 3, we propose a hierarchical structure for TP-GMM, shown in Algorithm 4. Demonstrations from each situation are encoded in separate TP-GMMs (**Π**_*n*_) in the training step. In the test step, the new situation is maximally matched with each demonstrated situation (the arg min step in the **for** loop), and we select the overall best match *n*^⋆^ for generalization in the DECODE function if the matched result *d*_*n*^⋆^_ is below a prescribed threshold. If the threshold is exceeded, new demonstrations should be requested. This process serves as a gating function, similar to the one in [17]. Finally, the Gaussian clusters in the generated world-frame GMM **π**^⋆^ are inversely morphed with *α*_*n*^⋆^_^−1^ to ensure that the final GMM **π** is compatible with and applicable to the actual, desired test situation **p**_test_. Note that the arg min step in the **for** loop in Algorithm 4 can be solved analytically, because of the linear operations on the task parameters and the L2-norms.

#### 2.2.5. Validation of HS in Simulation

We conducted simulations to empirically validate the hierarchical structure and compare its performance with vanilla TP-GMM (VT). The simulation procedure is detailed in Algorithm 5. We used *N*_train_ = 2, 3, ⋯, 10 and *M*_test_ = 100 to explore how test performance changes with an increasing number of demonstrated situations. We repeated SIM 20 times for each value of *N*_train_ so that we could extensively sample both training and test situations. We used *d*_threshold_ = ∞ with HS to disable new demonstration requests because VT cannot preemptively stop execution. Finally, we used three different task configurations of start, goal, and via frame sampling ranges to verify the hierarchical framework's performance.

**Figure 6:**
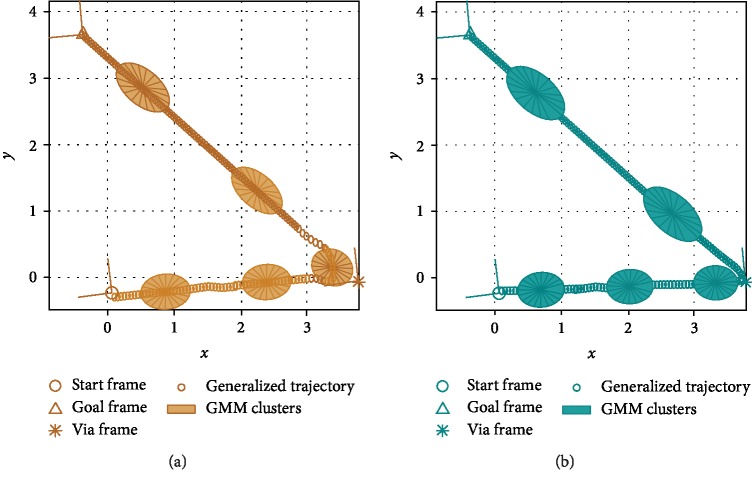
Generalized trajectories for a test when three demonstrated task situations are available: (a) vanilla TP-GMM (VT); (b) hierarchical structure (HS).

Figure 6 shows sample generalized trajectories for a test with three demonstrated task situations. We used EVAL in Algorithm 5 to calculate *generalization errors* as the squares of the distances between the first generalized trajectory point and the start frame, the last trajectory point and the goal frame, and the 100th trajectory point and the via frame. These three pairs of points should coincide for a trajectory that was perfectly generated according to the original demonstration strategy, yielding zero error.

**Figure 7:**
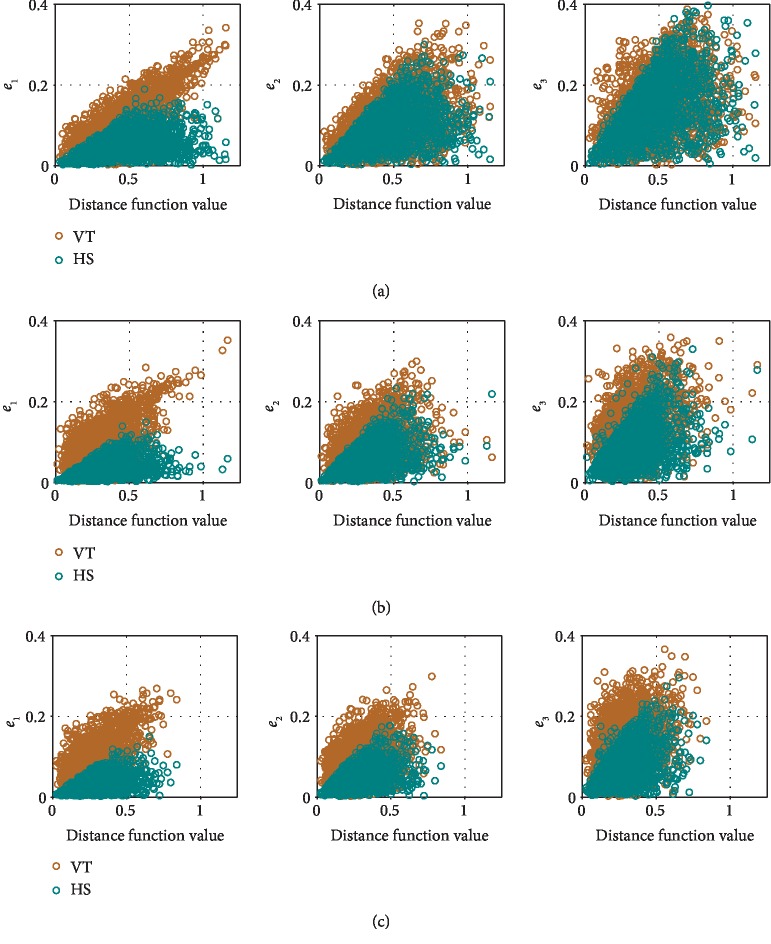
Generalization errors against distance metric when different numbers of situations were demonstrated during training: (a) with two situations; (b) with five situations; (c) with eight situations.

*(1) Distance Function as a Performance Estimator*. For the task configuration shown in Figure 4, Figure 7 shows the accumulated generalization errors against the corresponding distance function values. For brevity, we show results only when two, five, or eight situations were demonstrated. The general trend that the upper bounds of the generalization errors are roughly linearly proportional to the distance function value is true for all numbers of demonstrations. In other words, the distance value can serve as a cautious estimator of generalization errors so that if the user needs to maintain a minimum level of performance (maximum allowed generalization error), he or she can prevent task execution if the distance value is above a certain threshold and can instead provide more demonstrations to the robot.

**Figure 8:**
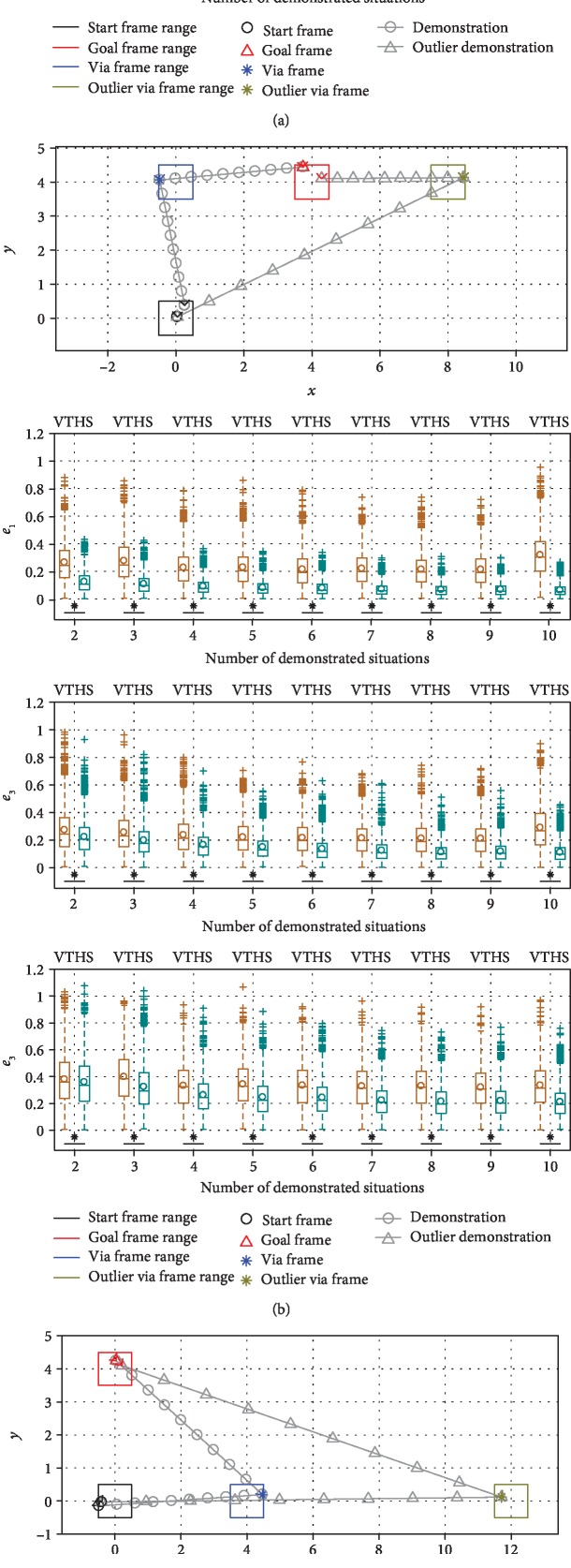
Comparison of generalization errors when different numbers of situations were demonstrated. The robot behavior abbreviations shown at the top stand for vanilla TP-GMM (VT) and hierarchical structure with TP- GMM (HS). Top panels show task configurations and samples of task situations, where each task frame was sampled from the specified range. When ten situations were demonstrated, the via frame of the tenth situation was drawn from an outlier range. Panels in the bottom three rows show boxplots of error metrics with different numbers of demonstrated situations in the corresponding task configurations. The center box lines represent the medians, and the box edges are the 25th and 75th percentiles. Circles show mean values. An asterisk and a horizontal line below boxplots show that the mean error from HS is significantly lower than the mean error from VT with *p* < 0.001.

*(2) Generalization Performance*. Figure 7 shows that HS achieved better performance (lower generalization errors) than VT. The accumulated generalization errors are shown for a few different task configurations in Figure 8 to fully verify that observation. It can be seen that as the number of demonstrated situations increases from two to nine (we explain the results with ten situations in the next point), generalization errors become lower for both VT and HS. Nevertheless, HS almost always has significantly better performance than VT.

**Figure 9:**
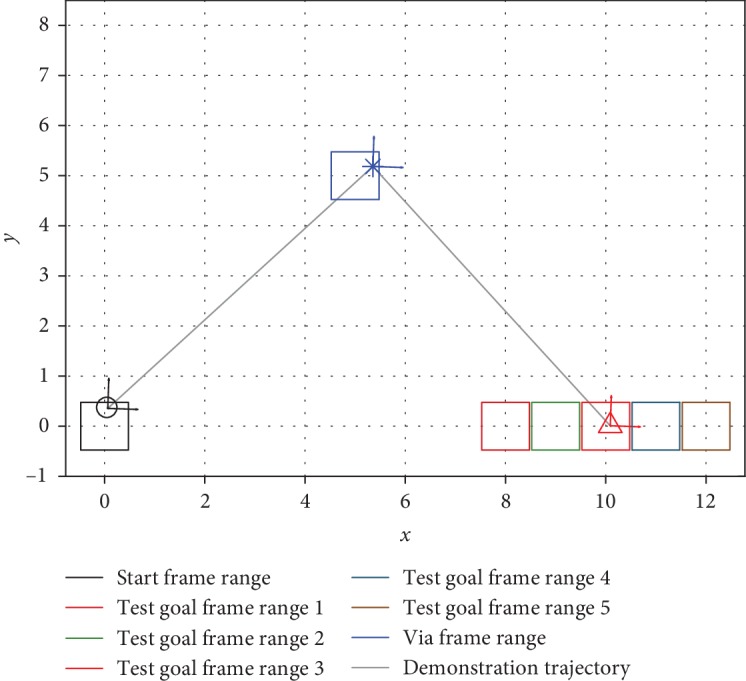
The test task configurations to evaluate interpolation/extrapolation performance. Note that the test goal frame range 3 is the same as the training goal frame range, so it evaluates interpolation performance.

**Figure 10:**
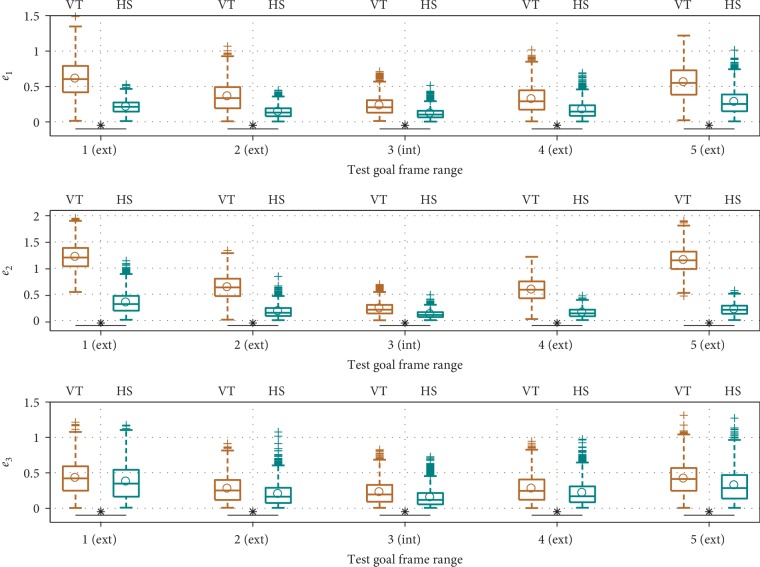
Performance comparison during interpolation (int) and extrapolation (ext). An asterisk and a horizontal line below boxplots show cases where the mean error from HS is significantly lower than VT with *p* < 0.001.

To showcase the difference in performance during interpolation/extrapolation, we performed an additional test where the training demonstrations were generated from the task configuration shown in Figure 4, while the goal frame ranges of test task configurations may be closer to or farther away from the start frame range, as shown in Figure 9. Because TP-GMM utilizes affine transformations as task parameters as opposed to scalar values or position vectors, we chose these test task configurations to indicate that the test task parameters could be drawn from the same distribution as the training task parameters (interpolation, test goal frame range 3) or from different distributions (extrapolation, test goal frame ranges 1, 2, 4, and 5). The simulation procedure was the same as Algorithm 5, and we used *N*_train_ = 5 and *M*_test_ = 100. The accumulated generalization errors are shown in Figure 10. It can be seen that performance was the best for both VT and HS during interpolation, and performance decreased more severely for heavier extrapolation (test goal frame ranges 1 and 5). Nevertheless, HS consistently achieved better results than VT in all configurations.

*(3) Effect of an Outlier Situation*. We mentioned that TP-GMM can be seen as a regression algorithm, with the task parameters as independent variables and the trajectories as dependent variables. Therefore, we explored the effect of having outlier training data in TP-GMM when there were ten demonstrated situations in the simulation procedure: the tenth situation had a different sampling range for the via frame, as shown in the top panels of Figure 8. It can be seen that VT suffered from this single outlier: compared to results with nine and sometimes even two demonstrated situations (see *e*_1_ in Figure 8(a) and *e*_2_ in Figure 8(c)), average generalization errors increased when demonstrations from ten situations were available, even though more training data is often assumed to improve test performance. On the other hand, HS was not affected by the outlier and maintained the same level of performance, because task parameters were used to first filter the training data, and only the most similar situation was used in generalization.

Note that although the outlier situations differ from the majority of the training data from the model's point of view, they may still be of interest to users and hence should be learned by the robot. Therefore, it may be more sensible for vanilla TP-GMM to encode the different situations in a separate model so that the robot can handle the outlier situations without affecting performance for the regular situations. However, to the best of our knowledge, allowing a robot to automatically determine when to create a new model is still an open problem regarding TP-GMM, precisely because the strengths of TP-GMM include handling data from varied task situations.

**Figure 11:**
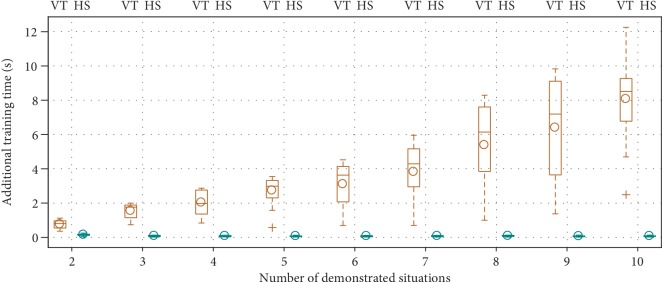
Additional time spent to train TP-GMMs increased as more demonstrated situations became available for VT but remained low for HS.

*(4) Training Time*. Another advantage that HS offers is reducing computational load when demonstrations from new situations become available gradually. The VT-TRAIN function always encodes all demonstrations together, which means previously encoded demonstrations have to be stored for reuse later. On the other hand, because HS-TRAIN encodes each situation in a separate TP-GMM, only new data needs processing when it becomes available.  Figure 11 shows the time spent only in training TP-GMMs as the number of available situations increases in the simulation procedure: HS consistently took little time to encode new data, while VT had to spend more time encoding everything.

Note that here we stored all demonstrations and computed a new TP-GMM every time for VT, instead of using one of the incremental TP-GMM methods in [23] because of the following reasons. First, the generative technique in [23] does not save computation time compared to VT because it samples trajectories using the existing model to represent previously encoded trajectories, which are then encoded with new trajectories to form a completely new model. Furthermore, performance may suffer because sampled trajectories are used in the new model instead of actual demonstrations. Second, the model addition technique in [23] will also take strictly more time than HS because it encodes new demonstrations in a new model like HS and then has to concatenate and optimize the previous and the new models together. Third, the direct update technique [23] assumes that the old demonstrations and the new ones are drawn from the same distribution, which is problematic because we sample from a relatively large number of task situations or even an outlier situation.

The advantage of reduced computational load is also true when removing demonstrations. In this example, HS can identify the outlier in the 10 demonstrated situations, because it was never selected for generalization, and thus, the corresponding TP-GMM could be deleted from the robot's database without affecting performance. In contrary, there is no inherent method in VT to identify the outlier, and even if a human operator identifies the outlier situation to be deleted, a new TP-GMM has to be trained from scratch to recover the performance of the nine remaining inlier demonstrations. Incremental methods such as [15, 23] do not seem to consider removing demonstrations.

#### 2.2.6. Comparison Summary between HS and VT

Compared to vanilla TP-GMM, the proposed hierarchical structure has higher complexity because it encodes a separate TP-GMM for each demonstrated task situation and has several utility functions to compare and morph test situations against demonstrated ones. However, this structured approach enables a robot to differentiate between the demonstrated situations and select the most similar experience for trajectory generalization. As a result, the robot can halt autonomous execution if its expected performance is lower than a predefined threshold. In contrast, VT does not offer these abilities. Because HS selectively uses demonstrations during generalization, test performance can also be improved via situation-specific manipulation of trajectories using the morphing functions. Moreover, it is possible to include a wide variety of task situations in the same HS framework without decreasing performance, while VT may lose situation-specific information because it effectively averages all demonstrations. Lastly, new demonstrations can easily be added to the skill library by creating a new TP-GMM, and poor or no-longer-wanted demonstrations that are already encoded can easily be removed with the hierarchical structure.

Special care needs to be considered when defining the utility functions in HS. For tasks such as object movement, Cartesian distances between corresponding task frames can readily be used in the distance function because they utilize the same information that TP-GMM considers. As for the morphing functions, our example validates the effectiveness of the hierarchical structure even with their simple and intuitive definitions. Other approaches, such as using reinforcement learning to optimize task parameters [25], may be used as well at the expense of additional design effort and computational load.

### 2.3. Experiment Validation on Real Hardware

We tested our proposed learning structure using a Willow Garage PR2 in the real collaborative object-manipulating task shown in Figure 1. The PR2 has two mirrored arms, each with four revolute arm joints and three revolute wrist joints. We collected demonstrations for three different task situations and conducted a human-subject study to validate the generalization performance of the proposed hierarchical structure in this task. Fifteen adults participated in the study, each completing the collaborative object-manipulation task under various task situations, experiencing the demonstration process, and filling out questionnaires to evaluate their interactions with the robot. The Penn IRB approved all experimental procedures under protocol 829536. Subjects gave informed consent and received no compensation for participating.

#### 2.3.1. Task Definition

The left arm of the PR2 robot holds an object (a rigid rectangular plate) together with the human partner; the robot and the participant collaborate to move the object from a start position to a goal position while avoiding an obstacle. The plate has a mass of 0.77 kg and a size of 0.30 m by 0.20 m by 0.01 m, and the obstacle is a slightly tapered plastic cylinder with a top radius of 0.23 m, as shown in Figure 1. The minimum and maximum distances between the robot end-effector at robot-shoulder height and the shoulder joint are about 0.4 m and 0.82 m, so the size of the obstacle is significant when compared to the robot's workspace. Thus, the robot's trajectories during collaborative movement need to make sense for the human partner for the task to be successful.

#### 2.3.2. Demonstrating Procedure

When collecting demonstrations, we used the PR2's right arm as the master and its left arm as the slave in bilateral teleoperation [26]. The teacher guided the master to help the human partner accomplish the desired task with the slave arm, therefore directly feeling the motions and limits of the robot arm, similar to how demonstrations are done in kinesthetic teaching. Demonstration recordings included the Cartesian position of the slave's wrist center calculated from forward kinematics. Force feedback between the master and the slave was achieved through a joint-level proportional-derivative (PD) torque controller, and therefore, no force or torque sensor was required.

For the master-side wrist joints, an additional virtual fixture [27] was applied to help the teacher control the robot's hand orientation. The virtual fixture torques were calculated using a PD controller with zero desired velocity:

(5) $$\begin{array}{c} \tau_{i,\text{vf}}=K_{p}\left(q_{i,\text{vf}}-q_{i,m}\right)-K_{d}{\dot{q}}_{i,m}, \end{array}$$

where *q*_*i*,*m*_ are the desired wrist joint angles for the virtual fixture. In the current work, we used the virtual fixture to constrain one degree of freedom of the gripper orientation: the desired center axis of the gripper frame was constrained to be horizontal in the world frame, and *q*_*i*,vf_ were found using inverse kinematics. The virtual fixture could also be used to satisfy task-specific requirements by choosing a different desired gripper orientation, e.g., when the carried object needs to be tilted to go through a doorway.

#### 2.3.3. Training Procedure and Robot Controller

The task frames were defined to include positions **r**_*p*_ and orientations **R**_*p*_ of the start (*p* = 1), the goal (*p* = 2), and the obstacle (*p* = 3). When collecting demonstrations, we used forward kinematics to determine the start and the goal poses, and we calculated the obstacle's pose by making the robot's end-effector touch the edge of the cylindrical obstacle along its radial direction and adding an offset of the cylinder's radius. Because the start, goal, and obstacle frames may have different orientations in each task situation, we expanded the previously listed distance function (Algorithm 2) to iteratively align all task frames, as shown in Algorithm 6.

We assumed the existence of a desired trajectory corresponding to each task situation. Given the robot's wrist trajectories (**x**) for a situation of interest, we resampled all trajectories to *L* data points based on trajectory length and used standard GMM/GMR to generate an *average trajectory* (**x**_avg_) in the world frame for that task situation. We then derived a *desired trajectory* for the task situation: $x_{\text{des}}=\left[\begin{array}{cc} {\left\{x_{\text{avg},l}\right\}}_{l=l_{d}+1}^{L} & x_{\text{interp}} \end{array}\right]$, where *l*_*d*_ serves as a look-ahead variable to make the robot appear more active during execution and **x**_interp_ is a linearly interpolated trajectory between the last average trajectory point (**x**_avg,*L*_) and the goal of the task situation (**r**_2_) in *l*_*d*_ steps. In the current work, *L* = 500 and *l*_*d*_ = 50. In the experiment, **x**_avg,*L*_ was typically close the goal point, and thus, **x**_interp_ generally was a short line segment connecting **x**_avg,*L*_ to the exact goal location.

We chose to learn TP-GMMs that use the robot's 3D wrist center position as the input and the desired 3D trajectory point calculated from above as the output: **ξ**^*𝒾*^ = **x** and **ξ**^*ℴ*^ = **x**_des_. Consequently, the task parameters were defined as

(6) $$\begin{array}{c} A_{p}=\left[\begin{array}{cc} R_{p} & 0\\ 0 & R_{p} \end{array}\right] \end{array}$$

and $b_{p}={\left[\begin{array}{cc} r_{p}^{⊤} & r_{p}^{⊤} \end{array}\right]}^{⊤}$, *p* = 1, 2, 3. We chose to use position rather than time for parameterization to increase robustness [28] and eliminate the need for phase estimation (e.g., [29]) or dynamic time warping. In addition, we updated definitions of **T**′ and **T**^″^ in Algorithm 3 to accommodate 3D trajectories and the dimensions of our TP-GMM:

(7) $$\begin{array}{c} T^{\prime }=\left[\begin{array}{cc} I+\alpha T-T & 0\\ 0 & I+\alpha T-T \end{array}\right],\\ T^{\prime \prime }=\left[\begin{array}{cc} \left(\alpha -1\right)T & 0\\ 0 & \left(\alpha -1\right)T \end{array}\right]. \end{array}$$

We selected three different task situations to collect demonstrations. We collected five demonstrations for each situation to ensure that the variability of trajectories was captured. The collected demonstrations and their task parameters were encoded in VT-TRAIN and HS-TRAIN, and the TP-GMMs (**Π**_VT_, {**Π**_n_}_*n*=1_^3^) were tested and evaluated in the user study. We used the Bayesian Information Criterion (BIC) [30] to determine the number of Gaussian clusters *M* for each TP-GMM (15 clusters for **Π**_VT_ and 4 or 5 clusters for each **Π**_*n*_).

At test time, the PR2 robot could calculate a desired wrist position (**x**_des_) from its current wrist position at each time step using VT-TEST or HS-TEST, and a generic PD controller was used to generate the motor commands:

(8) $$\begin{array}{c} \tau =J^{⊤}\left(K\left(x_{\text{des}}-x\right)-B\dot{x}\right), \end{array}$$

where **J** is the Jacobian matrix of the position dimensions, and we chose **K** and **B** as diagonal matrices with 120 N m^−1^ and 10 N s m^−1^. We also included a passive mode where the robot provided only gravity compensation for the half of the object's weight, with

(9) $$\begin{array}{c} \tau =-\frac{1}{2}J^{⊤}mg, \end{array}$$

where *m* is the mass of the held object and **g** is the gravity vector, assuming that the robot and the human share the object's weight equally. This control mode was included so we could directly test whether the complexity of TP-GMM confers any benefits.

#### 2.3.4. User Study

**Figure 12:**
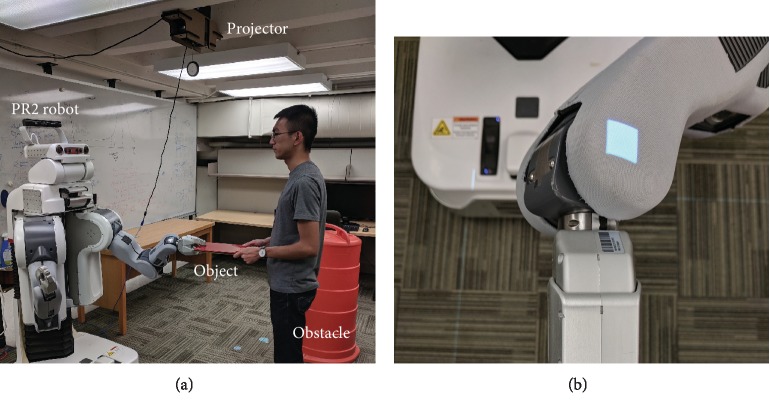
The experimental setup for the human-subject study. A projector helps the subject find the start, goal, and obstacle positions, and a square is projected on the PR2's wrist when the start position is reached.

We conducted a human-subject study to evaluate how the three described control algorithms affect task performance and how users perceive the robot behaviors in the collaborative movement task. Since the task situations were defined in the robot frame, we used a projector mounted on the ceiling to help participants identify and find the desired start, goal, and obstacle positions, as shown in Figure 12.

*(1) Participants*. Our participant pool consisted of 15 University of Pennsylvania affiliates, including undergraduate and graduate students, postdoctoral associates, and visiting researchers. Of the 15 participants, three were female and 12 were male, with ages ranging from 22 to 35 years (*μ* = 26.3, *σ* = 3.42).

*(2) Dependent Measures*. We recorded robot motions during each trial, and we used two quantitative measures to evaluate task performance: average trajectory length and average task completion time.

In addition, we used three questionnaires to evaluate the quality of the human-robot interactions during the task. First, the subject completed a Unified Theory of Acceptance and Use of Technology (UTAUT) survey [31] at the beginning and the end of the study. Results from the two surveys were compared to determine how interacting with the robot affected the general perception of subjects toward using the robot in everyday tasks.

Second, we adapted the questionnaire used in [32] and asked participants to answer the following questions on a 100-point scale from strongly disagree to strongly agree after each collaboration trial:

(Q1) The robot moved too fast

(Q2) The robot moved too slowly

(Q3) The robot had problems doing the task

(Q4) I felt safe when working with the robot

(Q5) I trusted the robot to do the right thing at the right time

(Q6) The robot and I worked well together

These questions sought to evaluate how subjects perceived the robot's behaviors and performance.

Third, a NASA-TLX survey [33] was administered after the participants experienced the process of providing demonstrations via teleoperation to gauge the workload of this interaction.

*(3) Procedure*. The human-subject study consists of two main components: collaborating with the robot and providing demonstrations. We chose to put the collaborating component first because it allows participants to become more familiar with the robot before demonstrating new movements.

**Figure 13:**
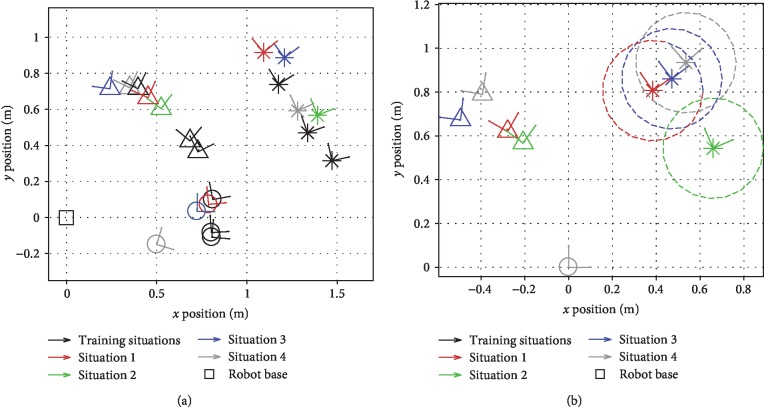
Task situations involved in the experiment. Circles represent the start frames, triangles the goal frames, and asterisks the obstacle frames. Dashed circles represent the boundary of the obstacle. Note that the start frames of situations 2 and 3 coincide. With respect to the three demonstrated situations, the *d*_*n*^⋆^_ values for these four common situations in Algorithm 4 with the definition in Algorithm 6 are 0.071 m^2^, 0.151 m^2^, 0.295 m^2^, and 0.398 m^2^: (a) training and the four common situations in world frame; (b) the four common situations aligned by their start frames.

**Figure 14:**
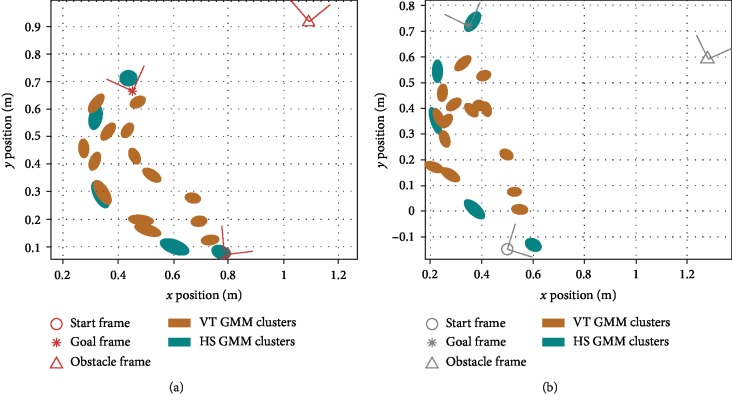
Generalized GMM clusters (**ξ**^*ℴ*^ dimensions) for two test situations from Figure 10: (a) situation 1; (b) situation 4.

The first component, collaborating with the robot, took place after the opening UTAUT survey. Participants were asked to collaborate with the robot to move the object from the start to the goal in five different situations. Four common situations, shown in Figure 13, were predetermined by the research team and were the same for all subjects, with situation 1 having the lowest and situation 4 having the highest *d*_*n*^⋆^_ value. These common situations were chosen to be somewhat close to the training situations so that both VT and HS should intuitively work well. They span a relatively large portion of the robot's workspace (Figure 13(a)) and have varied configurations (Figure 13(b)). Recall that we use TP-GMMs to encode data with **ξ**^*𝒾*^ = **x** and **ξ**^*ℴ*^ = **x**_des_.  Figure 14 shows the **ξ**^*ℴ*^ dimensions of generalized VT and HS GMM clusters for two example test situations. Situation 5 was chosen by each subject so that a large number of situations were sampled in the study.

Participants compared three different robot behavior modes: (P) passive with gravity compensation for the object, (VT) active with vanilla TP-GMM, and (HS) active with hierarchical structure and TP-GMM. Therefore, each participant evaluated the collaboration in 15 different combinations of task situation and robot behavior. For each situation, we asked participants to experience all three robot behaviors sequentially (completing two collaborations under each robot behavior), and then reexperience and rate each robot behavior. After the subject evaluated all three robot behaviors, a new situation was presented. This process repeated until all five situations had been shown. For each subject, the five situations were presented in a randomized order, and within each situation, the presentation order of the three robot behaviors was also randomized. Examples of human-robot interactions during the user study are shown in the supplementary video attachment.

Then, participants were presented with the second component of the study: providing demonstrations. The experimenter acted as the partner and moved the object with the robot, and the participant manipulated the robot's right arm to teleoperate the slave arm. Each participant experienced the demonstrating process for three to five minutes and then filled out the NASA-TLX survey. Finally, the participant filled out the closing UTAUT survey.

*(4) Hypotheses*. Based on the dependent measures, our main hypotheses for this experiment were as follows:

- The hierarchical structure (HS) will lead to better task performances, including shorter trajectory lengths and lower task completion times, compared to using vanilla TP-GMM (VT) and passive gravity compensation (P)
- Participants will be more satisfied with the robot when it uses the hierarchical structure (HS) to generate motion controls, compared to VT and P
- Given feedback after task execution in a wide variety of situations, the robot will be able to learn a decision boundary for when to ask for new demonstrations

## 3. Results

**Figure 15:**
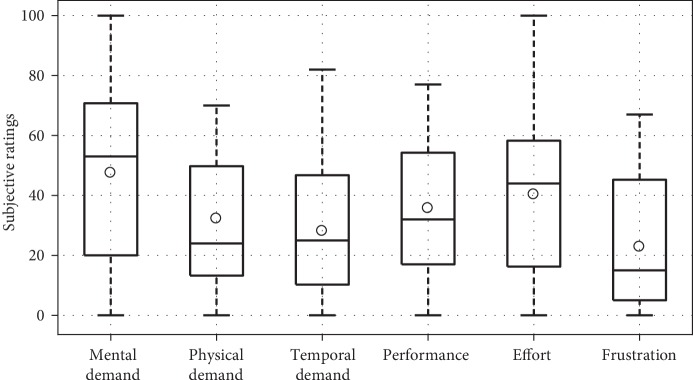
NASA-TLX ratings of the teaching procedure (lower is better). For the performance question, 0 indicates perfect and 100 indicates failure.

Figure 15 shows results from the NASA-TLX survey that subjects completed after providing demonstrations. The median value for “How mentally demanding was the task?” was 53 out of 100, and the median values for the other five questions were all below 50, indicating that the subjects perceived teaching by teleoperation as a low-to-moderate-effort task.

**Figure 16:**
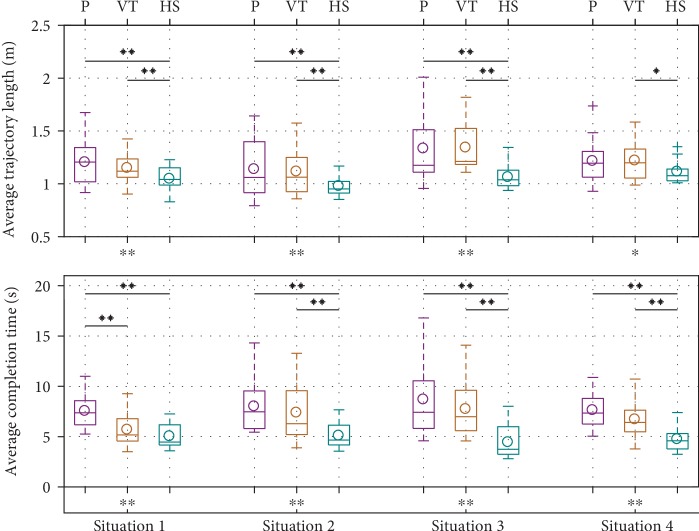
Comparison of quantitative measures for the four common task situations (lower is better). Asterisks under the *x*-axes show significant differences in each group, and asterisks and horizontal lines above boxplots show pairwise significant differences. ^∗^*p* < 0.1; ^∗∗^*p* < 0.05.

Objective performance at the collaborative movement task was determined by calculating the two quantitative measures of average trajectory length and average task completion time for the four common task situations. Results are shown in Figure 16. One-way repeated measures analysis of variance (ANOVA) was used to determine whether the differences in these measures between the three behavior modes under the same task situation were significant. If there was, a Tukey-Kramer post hoc multiple comparison test was conducted to determine which robot behaviors produced significantly different ratings. It can be seen that with the hierarchical structure, traversed trajectories were significantly shorter and took significantly less time for almost all situations, while VT reduced average completion time compared to passive gravity compensation only in test situation 1, which is the most similar to the demonstrated situations.

**Figure 17:**
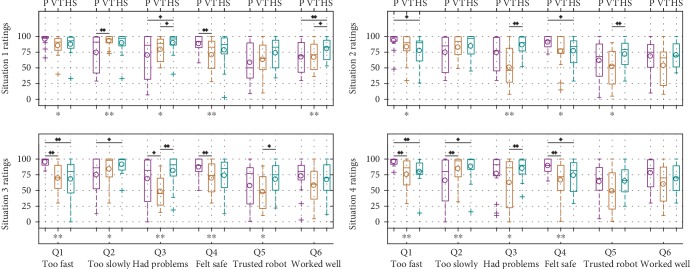
Comparison of subjective ratings (higher is better). Questions Q1–Q6 are detailed in Section 2.3.4. ^∗^*p* < 0.1; ^∗∗^*p* < 0.05.

**Figure 18:**
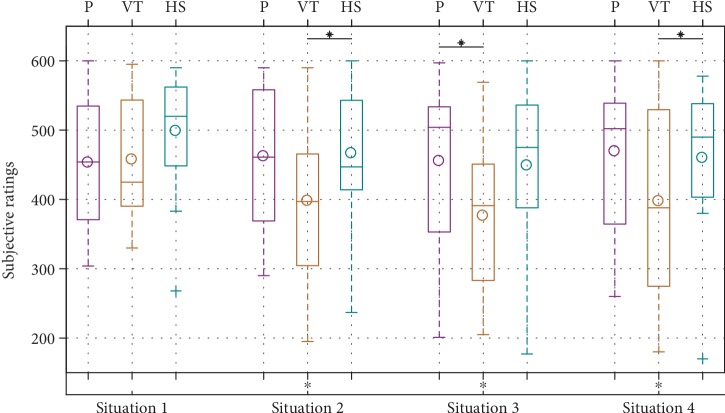
Comparison of sums of subjective ratings (higher is better). ^∗^*p* < 0.1.

Results from the subjective ratings under the four common task situations are shown in Figure 17. Plotted ratings of Q1, Q2, and Q3 were subtracted from 100 so that a higher rating is better for all questions. The same procedure used for the quantitative measures was used to determine significant differences. It can be seen that with passive gravity compensation, the robot almost always appeared significantly slower (Q1, Q2) and safer (Q4). No significant differences in perceived pace and safety were found between the two active modes. Compared to vanilla TP-GMM, the hierarchical structure appeared to have significantly fewer problems doing the task (Q3) in all situations. In situations 2 and 3, participants had more trust in the robot doing the right thing at the right time (Q5) with the hierarchical structure than vanilla TP-GMM. Finally, participants felt they worked better with the robot (Q6) with the hierarchical structure than the other two modes in situation 1.  Figure 18 shows the sums of these ratings. Vanilla TP-GMM generally had the lowest rating sums. Significant differences were found between the hierarchical structure and vanilla TP-GMM in situations 2 and 4 as well as between passive gravity compensation and vanilla TP-GMM in situation 3.

**Figure 19:**
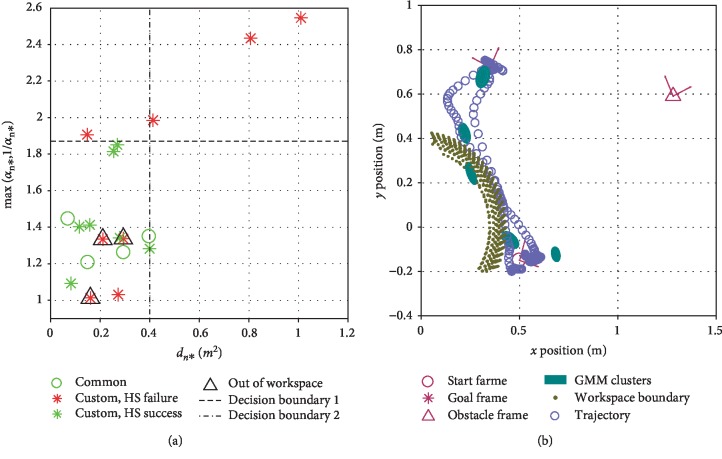
Learning when to request new demonstrations. (a) Rated success with the hierarchical structure. Two example decision boundaries are given. (b) An example with some GMM clusters outside of the workspace.

Results from the subjective ratings under the participant-selected situations were used to validate the distance function as a performance estimator in the collaborative task. To acquire labels of successful/failed execution, we defined the following criterion: an HS success has a subjective rating sum that is greater than 80% of the largest sum of ratings for any control mode under any task situation from the corresponding subject.  Figure 19(a) shows the classification results using the above criterion, where we additionally include a second feature dimension (max(*α*_*n*^⋆^_, 1/*α*_*n*^⋆^_)), which is calculated from the distance function in HS-TEST and represents the degree of stretching or compression in the morphing functions. We also plot the common situations in Figure 19(a) for completeness; these are manually labeled as successes with the hierarchical structure, because there were significant advantages in the quantitative measures and some significant advantages in the subjective ratings. Three of the custom situations for which the hierarchical structure was rated as a failure are manually marked as being out of workspace, because the obstacle was placed so close to the robot that some GMM clusters of the hierarchical structure policy were outside of the robot's workspace. As a result, the robot arm would become stuck when it first reached the workspace boundary following the control policy, and the robot arm would then slide along the workspace boundary and appear less smooth to participants. This behavior at least partially caused these three poor ratings; an example is shown in Figure 19(b).

Note that each participant tested only one custom situation and hence contributed one data point to Figure 19(a). The distribution might look different if a single participant did all of the tested situations, and it might change for different participants. Nevertheless, not counting the out-of-workspace situations, one could simply place a decision boundary for when to ask for new demonstrations at max(*α*_*n*^⋆^_, 1/*α*_*n*^⋆^_) = 1.87 with one misclassified task situation or at *d*_*n*^⋆^_ = 0.4 m^2^ with two misclassified data points.

**Figure 20:**
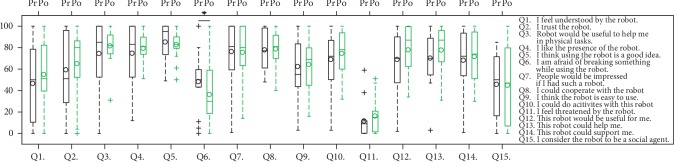
UTAUT ratings from prestudy (Pr) and poststudy (Po) surveys. ^∗^*p* < 0.1.

Figure 20 shows the ratings from the UTAUT questions. Paired sample *t*-tests were used to determine whether the differences between the means of these ratings were significant. A significant difference was found for the question “I am afraid of breaking something while using the robot,” indicating subjects were less afraid of breaking something after the study. No significant differences were found for other questions.

## 4. Discussion

The results from the user study provide strong support for our first hypothesis: the significant differences in the quantitative measures in favor of HS indicate that explicitly reasoning about task situations and generalizing over selected demonstrations could lead to significantly better trajectories and hence better task performance. In comparison, although VT also uses task parameters to annotate and process the demonstrations in ENCODE and DECODE, it does not differentiate between demonstrated situations and thus cannot apply situation-specific trajectory and model morphing to better accommodate the test situation. As a result, VT falls short of HS in task performance. Passive gravity compensation offers little help to users and leads to the longest trajectory lengths and task completion times.

Hypothesis 2 centers on user perception of the three tested robot control modes. In the subjective ratings, HS was rated to be significantly less problematic (Q3) than VT in all situations, indicating that users were able to differentiate the two modes and preferred HS. Furthermore, HS achieved significantly higher ratings than VT for trust (Q5) and working well together (Q6) in particular situations. The sum of the subjective ratings generally favored the hierarchical structure and passive gravity compensation, with the former being perceived as fast and effective and the latter as slow and safe. Thus, we conclude that the subjective ratings support our second hypothesis.

Results from the custom situations provide some support for our third hypothesis, which stated that the robot would be able to learn a decision boundary for when to ask for new demonstrations in the object movement task. In the particular instance of Figure 19(a), only one situation would have been misclassified by the robot with decision boundary 1. We think that predicting the performance of generalized behaviors is a critical component of LfD, since demonstrations are typically available only for a small subset of task situations and robot designers often cannot test every possible one. In addition to uncertainty- or confidence-based methods, TP-GMM offers unique opportunities in this effort because it utilizes the task parameters that contain additional information about the demonstrations and also enhance generalization capabilities. As for the custom situations with GMM clusters out of the robot's workspace, approaches to the Procrustes problem [34] could have been used to select an initial pose for the robot base before autonomous execution.

The NASA-TLX survey and the pre- and poststudy UTAUT surveys were not used to evaluate our hypotheses, but they provide some insights on the proposed approach. When subjects provided new demonstrations using our kinesthetic teleoperation method, they indicated that the heaviest workload was mental, most likely because the robot's motion was a mirror image of the demonstrated motion. In the pre- and poststudy UTAUT surveys, subjects became less afraid of breaking things when using the robot, suggesting that the human-robot interactions during the study had a slight positive effect on their opinions about the robot. Importantly, the participants experienced multiple robot behavior modes and multiple task situations where the robot might have worked well or poorly, which may explain the lack of significant changes in other questions.

## 5. Conclusions

The hierarchical structure proposed in this paper enables robots to additionally reason about task situations when utilizing TP-GMM. We showed that task performance can be improved in both interpolation and extrapolation scenarios and that computational load can be reduced with the hierarchical structure in simulation. We then showed that a robot can use the hierarchical structure to collaborate better with people in a real object movement task, also learning a decision boundary for when to ask for new demonstrations.

## Figures and Tables

**Algorithm 1:**
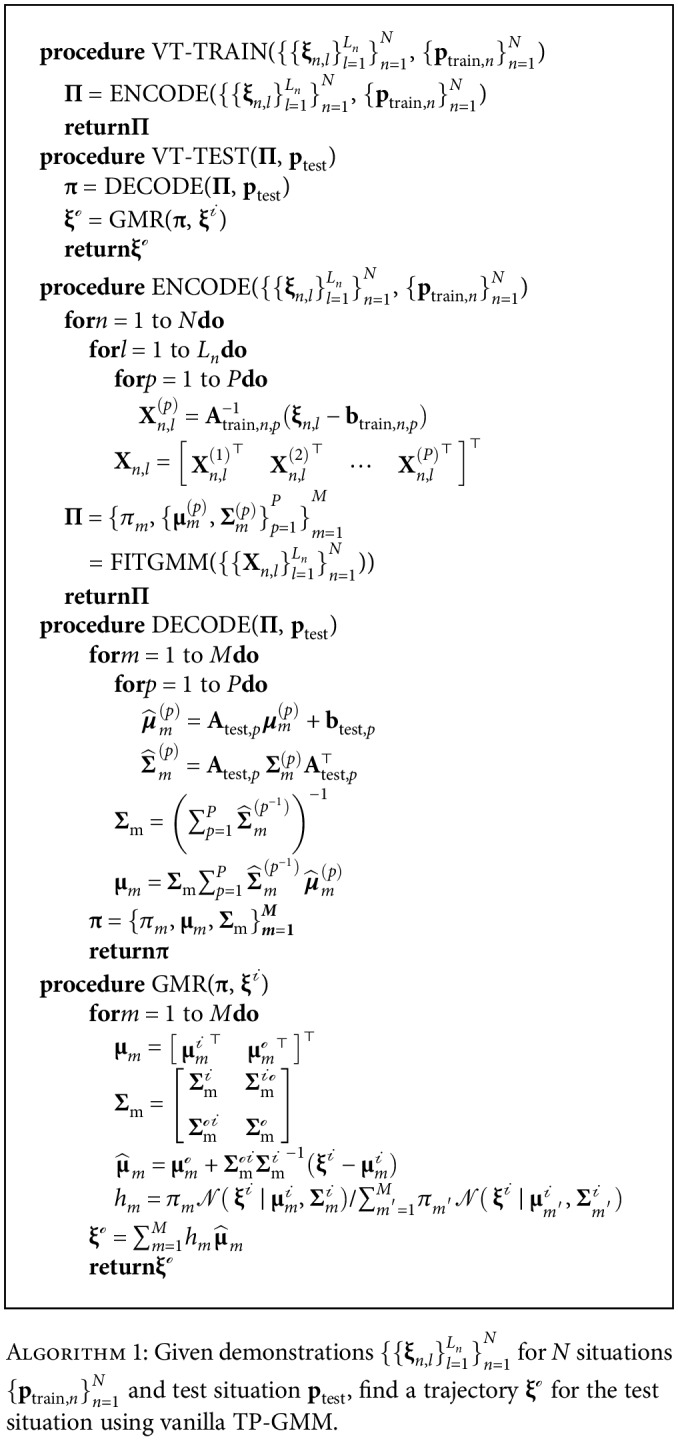
Given demonstrations {{**ξ**_*n*,*l*_}_*l*=1_^*L*_*n*_^}_*n*=1_^*N*^ for *N* situations {**p**_train,*n*_}_*n*=1_^*N*^ and test situation **p**_test_, find a trajectory **ξ**^*ℴ*^ for the test situation using vanilla TP-GMM.

**Algorithm 2:**
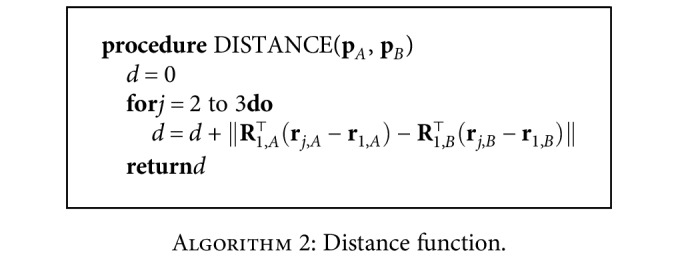
Distance function.

**Algorithm 3:**
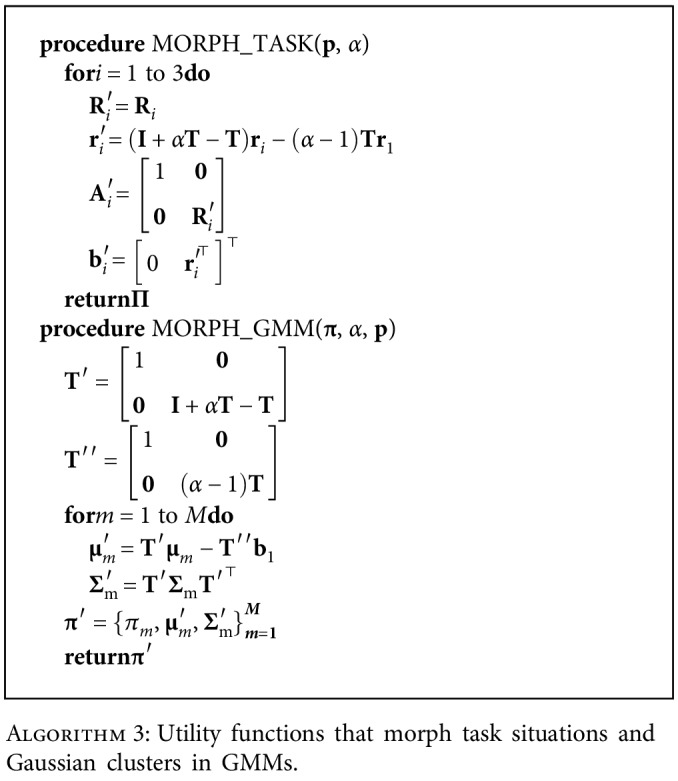
Utility functions that morph task situations and Gaussian clusters in GMMs.

**Algorithm 4:**
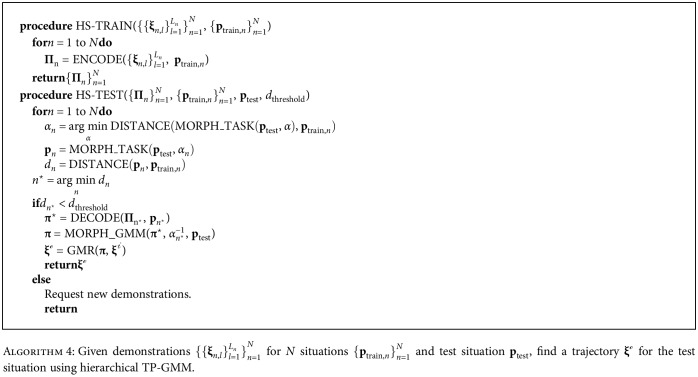
Given demonstrations {{**ξ**_*n*,*l*_}_*l*=1_^*L*_*n*_^}_*n*=1_^*N*^ for *N* situations {**p**_train,*n*_}_*n*=1_^*N*^ and test situation **p**_test_, find a trajectory **ξ**^*ℴ*^ for the test situation using hierarchical TP-GMM.

**Algorithm 5:**
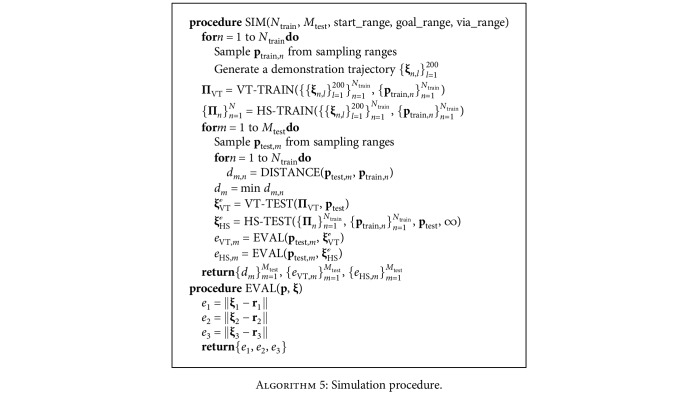
Simulation procedure.

**Algorithm 6:**
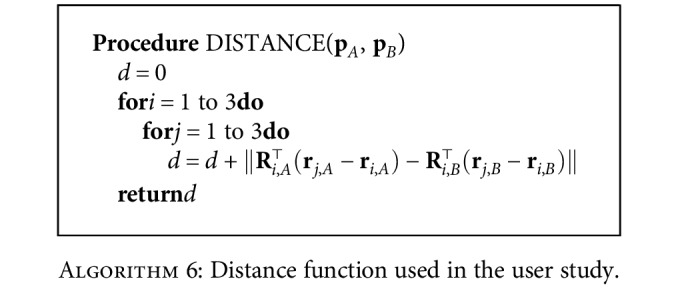
Distance function used in the user study.

## Data Availability

The data used to support the findings of this study are available from the corresponding author upon request.

## Supplementary Materials

Supplementary Materials We show three example trials with typical interactions encountered in the human-subject study, where the start and goal positions were the same. The robot said “start” at the beginning of each trial, and it said “reached goal” when the object was successfully moved to the prescribed goal position. In example trial 1, the robot successfully and quickly finished the task with the subject. In example trial 2, the robot's trajectory did not end at the goal location, and the human partner had to correct it by overpowering the robot. In example trial 3, the robot additionally did not make enough room for the partner to walk by the obstacle easily, so the subject slightly rotated his body when walking towards the goal.
